# Salivary Biomarkers Analysis and Neurobehavioral Assessment in Nurses Working Rotation Shifts: A Pilot Study

**DOI:** 10.3390/ijerph20075376

**Published:** 2023-04-03

**Authors:** Silvia Vivarelli, Sebastiano Italia, Michele Teodoro, Manuela Pollicino, Carmen Vitello, Annalisa De Vita, Angela Alibrandi, Chiara Costa, Concettina Fenga

**Affiliations:** 1 Department of Biomedical and Dental Sciences, Morphological and Functional Imaging, Section of Occupational Medicine, University of Messina, 98125 Messina, Italy; 2 Department of Economics, University of Messina, 98125 Messina, Italy; 3 Department of Clinical and Experimental Medicine, University of Messina, 98125 Messina, Italy

**Keywords:** shift work, salivary biomarkers, cortisol, alpha-amylase, melatonin, occupational health, neurobehavioral alterations, work-related stress, sleep quality

## Abstract

Currently, about one in five workers is employed in night shift work in Europe. Shift work including nighttime hours is essential in several activities, especially the healthcare sector. Importantly, night working may be associated with the occurrence of sleep disorders or work-related stress, both potentially augmenting the risk of errors and accidents at work. This study aims to examine the presence of neurobehavioral alterations that can be a consequence of shift working and concurrent misalignment of the sleep times and circadian rhythms. Nurses (*n* = 102) employed at a University Hospital located in North-Eastern Sicily, Italy, voluntarily participated in this pilot study. During medical surveillance, morning and evening salivary samples were collected, and seven psychodiagnostics questionnaires were administered to all the subjects. On one hand, the salivary levels of stress-related biomarkers (cortisol and alpha-amylase) and a circadian biomarker (melatonin) were evaluated. On the other hand, several neurobehavioral features were assessed, including depression, anxiety, work-related, and sleep issues. Interestingly, a positive relationship between salivary morning cortisol and depression scale, as well as a negative relationship between salivary morning alpha-amylase and work ability scale, were observed. Based on these results, the integration of subjective questionnaire outcomes and objective salivary biomarker quantification can help to identify workers with increased susceptibility to developing neurobehavioral alterations. This approach may contribute to ameliorating preventive strategies towards sensitive categories, such as nurses working rotation shifts.

## 1. Introduction

Shift work is considered any schedule that falls outside the canonical working hours (7 a.m.–6 p.m.), and it often includes night shift work, defined as work carried out for at least 3 h of a daily shift (or a certain proportion of yearly working time) over a period of 7 h, including the time from midnight to 5 a.m. (Directive 2003/88/EC, articles 2.4 and 2.5) [1]. Following a misalignment in sleep time and circadian rhythms, night shift work poses significant risks to workers employed in the healthcare system [2]. In fact, it can alter circadian rhythms, therefore affecting sleep quality [3]. This condition can lead to a so-called “shift work disorder”, characterized by complaints of excessive sleepiness during daytime and/or insomnia, due to the fact that healthcare workers might fail to adapt to work during normal sleep times [4]. Additionally, stress issues including depression, anxiety, imbalance between effort and reward at work, and general loss of motivation to accomplish working tasks (work ability), may represent adverse consequences related to night working, especially in the healthcare sector [5]. Indeed, shift work sleep disorders and stress issues are tightly interconnected, especially in nurses, often suffering from extended working hours, heavy workloads, overtime, and short rest periods between shifts [6]. This may directly affect not only nurses but also the community because health unbalances could lead to not only decreased work performance, exhaustion, and possible burnout but also medical errors [7].

The management and prevention of such consequences and, hence, the protection of the wellbeing of nurses are key for the medical surveillance system [8]. In order to assess symptoms possibly linked to sleep alterations and/or arising stress, the administration of psychodiagnostics questionnaires plays a pivotal role. Nevertheless, questionnaires have the intrinsic limitation of being based on a subjective self-awareness, and therefore, they may lack objectivity [9,10]. For that reason, it could be very helpful to combine such tools with biological parameters that may objectively indicate the presence of either sleep disorder or work-related stress [3]. Saliva is increasingly employed for the screening and early diagnosis of several diseases. In fact, it contains a variety of biomolecules (including nucleic acids, metabolites, hormones, and immunoglobulins) [11]. Additionally, saliva sample collection is easy, cost-effective, and non-invasive, thus enhancing patients’ compliance. Many companies are currently investing in the development of reliable kits to detect and easily quantify potential salivary biomarkers for innovative precision medicine approaches [12]. Indeed, the current coronavirus pandemic situation elicited high emotional stress, especially in healthcare workers; hence, it has becomes advantageous to look for noninvasive biomarkers in order to keep stress levels adaptative and to reduce hazards for workers [13,14].

Both short-term (e.g., sleep deficiency, hormonal imbalance, and inflammation) and long-term (e.g., cardiovascular, metabolic, and mental diseases, and cancer) health consequences of shift work derive from the misalignment between the circadian rhythms and the timing of the sleep/wake cycle, which is controlled by the pineal hormone melatonin [15]. Salivary melatonin is a widely used circadian phase biomarker [16]. Night shift work may be associated with a drop in melatonin production and its circadian rhythm alteration due to interference with the pathways modulated by nocturnal light [17]. Physiologically, the circadian rhythms regulate hormonal secretion [18]. In particular, cortisol levels are high in the morning, reaching the peak thirty minutes after awakening and its lowest concentrations in the evening. This secretion pattern is essential for proper functioning of the human organism [19]. Conversely, chronic stress is linked to the stimulation of the hypothalamic–pituitary–adrenal axis, thereby causing an increase in both plasmatic and salivary cortisol concentrations [20,21]. Similarly, acute stress is correlated with the activation of the adrenomedullary sympathetic system, with a subsequent increase in epinephrine and norepinephrine levels, followed by a sudden increase of salivary alpha-amylase [22]. Both cortisol and alpha-amylase can reach saliva by passive diffusion; hence, they may be used as early indicators of stress [23].

In this context, the objective of this pilot study is to examine in a population of nurses working rotation shifts the potential linkages between salivary cortisol, alpha-amylase, and melatonin measurement and stress- and sleep-related questionnaires responses. This approach may help occupational physicians with the early identification of vulnerable individuals at higher risk of developing disturbances possibly linked to night shift work and circadian rhythm disruption.

## 2. Materials and Methods

### 2.1. Study Design and Population

**Figure 1:**
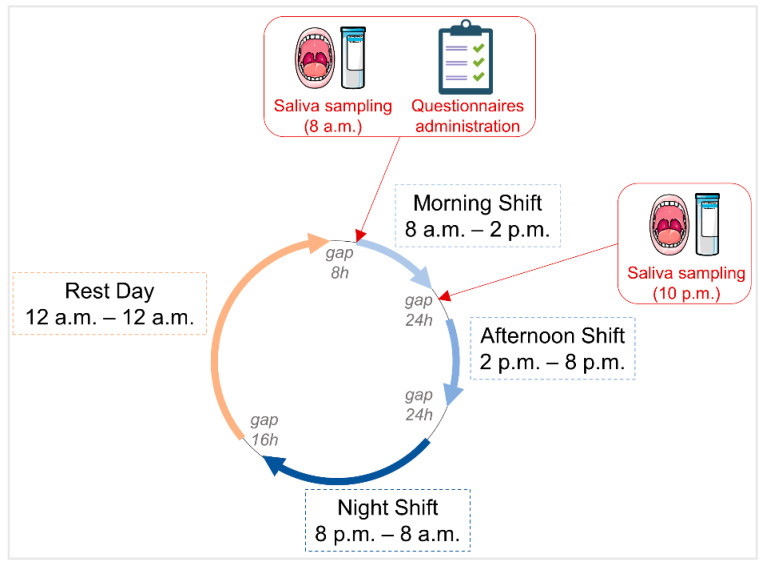
Shift schedule schematic and study procedure. Thick blue arrows represent clockwise work shifts (light blue, morning; medium blue, afternoon; dark blue, night); thick orange arrow represents the rest day. Gap lines between arrows represent non-working time. Red arrows show sampling timepoints during the procedure.

This study was carried out from October 2020 to January 2021 and from May to August 2021 at a University Hospital located in North-Eastern Sicily, Italy. Both males and females (respectively, *N* = 47 and *N* = 55) working rotating shifts participated in the study. In order to reduce confounding factors, subjects suffering from neurological and/or psychiatric conditions were excluded. All participants voluntarily agreed to participate in the study and did not receive any monetary compensation. All participants were night shift workers and followed a fast-rotating, 5-day-long, forward (clockwise) shift system, which is largely adopted as a standard schedule for hospital nurses, both in Europe and Japan [24]. In such a forward-rotating shift schedule, a morning shift is followed by an afternoon shift and a night shift, which is followed by a rest day (Figure 1). The human physiological sleep/wake cycle best adapts to the forward rotating shift [25].

According to the Italian legislation, nurses are required to undergo a compulsory medical surveillance [26]. On that occasion, a multidisciplinary team, composed by medical doctors from the Occupational Medicine Section of the University of Messina, with the cooperation of psychologists, clearly explained the aim of the investigation to the nurses, administered the validated psychodiagnostics questionnaires, and collected the salivary samples.

Additionally, during the medical surveillance, medical personnel collected information about sociodemographic characteristics, health status, lifestyle habits, and work-related factors. This information included gender, age, educational level, marital status, parental status, body mass index, systolic and diastolic blood pressure, heart rate, smoking habits, alcohol consumption, use of chronic medications, work injuries, and work seniority.

### 2.2. Salivary Sample Collection

Cryogenic tubes and Saliva Collection Aids (Item No. 5016.02, Salimetrics, State College, PA, USA) were used to collect saliva samples. The saliva collection aid employed in the study is based on a passive drool, representing a validated gold standard for saliva sampling. Salivary samples were collected at the beginning of the morning shift, two hours after awakening, at 8 a.m. (for morning cortisol, melatonin, and alpha-amylase analysis), and before the medical examination and administration of psychodiagnostics questionnaires. In addition, the nurses were instructed to collect a salivary sample with a second kit at 10 p.m. (for evening cortisol analysis; Figure 1). Twelve hours prior to saliva sampling, the subjects were asked to avoid foods with high content of sugar or high acidity and to avoid the use of nicotine, caffeine, and alcohol. To prevent saliva dilution, participants were required to rinse their mouth with water and to wait 10 min before sampling. Upon collection, all samples were immediately stored at −20 °C until further processing.

### 2.3. Salivary Enzyme Measurement

Cortisol, alpha-amylase, and melatonin were detected in salivary samples collected in the morning (8 a.m.). Evening cortisol was detected in salivary samples collected in the evening (10 p.m.). The salivary cortisol enzyme immunoassay kit (Item No. 1-3102), salivary alpha-amylase kinetic enzyme assay kit (Item No. 1-1902), and salivary melatonin enzyme immunoassay kit (Item No. 1-3402) were all purchased from the Salimetrics (Salimetrics, State College, PA, USA). Salivary biomarker quantifications were performed according to the manufacturer’s instructions.

Specifically, on the day of the assay, the saliva samples were thawed, vortexed, and centrifuged at 1500× *g* for 15 min to precipitate mucins. Hence, 25 µL or 100 µL of a saliva supernatant was used for cortisol and melatonin assessment, respectively, whereas 8 µL of 1:200 diluted saliva samples was used for alpha-amylase activity measurement. For cortisol and melatonin quantification, the samples were incubated with enzyme horseradish peroxidase (HRP) conjugate substrates and, after washing, with tetramethylbenzidine (TMB) substrate solution. Following the addition of an acidic stop solution, the optical density (OD) was read at 450 nm (plus additional wavelength correction reads at 490 nm for cortisol and at 620 nm for melatonin). For alpha-amylase activity, an enzyme substrate was added to the diluted saliva samples and, following immediate mixing, the OD at 405 nm was read after 1 min and after 3 min of incubation. All spectrophotometric measurements were performed using a Sinergy HT microplate reader (Bio-tek, now part of Agilent, Santa Clara, CA, USA). Each measured analyte was expressed either as concentration or enzymatic activity per unit of volume.

### 2.4. Neurobehavioral Assessments

#### 2.4.1. Beck Depression Inventory (BDI)

The Beck Depression Inventory (BDI) is a self-report depression scale widely used to measure different aspects of depression, such as affective, cognitive, motivational, and physiological. The questionnaire consists of 21 items ranked on a 4-point Likert scale from 0 to 3. Each item is provided four response choices according to the severity of the symptoms, ranging from an absence to an intense level of a specific symptom (e.g., sadness, discouragement, guilt, disappointment, and irritation) during the prior week. The total score is obtained by summing each item score. Total scores from 0 to 9 indicate normal mood, from 10 to 18 indicate minimal depression, from 18 to 29 indicate mild depression, and greater than 29 indicate severe depression [27,28].

#### 2.4.2. Hamilton Anxiety Scale (HAM-A)

The Hamilton Anxiety Scale (HAM-A) represents one of the first rating scales to measure the severity of perceived anxiety symptoms, both psychic anxiety (i.e., mental agitation and psychological distress) and somatic anxiety (i.e., physical complaints related to anxiety). It is a 14-item questionnaire, where each item is scored on a 3-point scale, and the total score is the sum of each score. A total score from 0 to 7 corresponds to an absence of anxiety; from 8 to 14 indicates mild anxiety; from 15 to 21 shows moderate anxiety; from 22 to 29 indicates severe anxiety; and finally, higher than 29 indicates extremely severe anxiety [29].

#### 2.4.3. Effort–Reward Imbalance (ERI)

Work-related stress may be identified as non-reciprocity or imbalance between high effort spent and low reward received. Additionally, overcommitment (or excessive personal motivation to work) may also increase the risk of stress-related adverse health outcomes. The Effort–Reward Imbalance (ERI) is a questionnaire measuring effort, reward, and overcommitment at work [30].

The Italian short version of the ERI questionnaire was administered to subjects of this study. The Italian ERI questionnaire is constituted of a total of 16 4-point Likert-scaled items (strongly disagree, disagree, agree, and strongly agree). The questionnaire contains three subscales concerning effort, reward, and overcommitment. The effort subscale is measured with three items, the reward subscale is measured with seven items, and the overcommitment subscale is measured with six items. The Siegrist algorithm allows for the subscale scores and the Effort/Reward (ER) ratio to be calculated. For ER = 1, the subject reports one effort for one reward, whereas for ER < 1, the subject reports less effort for each reward, and for ER > 1, the subject reports more effort for each reward [31,32].

#### 2.4.4. Work Ability Index (WAI)

The Work Ability Index (WAI) questionnaire is used in occupational medicine to assess the ability to work during workplace surveys and health examinations. The questionnaire is composed of seven items, including a self-evaluation of current work ability compared with the lifetime best or in relation to job demands, diagnosed illnesses and their link to estimated work impairment, sick leave during the past year, and mental resources. The total WAI score is categorized into poor (score 7–27), moderate (score 28–36), good (score 37–43), and excellent (score 44–49) [33].

#### 2.4.5. Morning Evening Questionnaire (MEQ)

The Morning Evening Questionnaire (MEQ) is composed by 19 multiple choice questions concerning morningness and eveningness tendencies, thereby defining the subject chronotype. This allows us to highlight which time of the day may be associated with higher alertness and what is the individual time preference for performing both physical and mental activities (i.e., night owls versus early birds). In accordance with the total score obtained with the MEQ, individuals may fall into one of the following categories: absolutely morning type (score 70–86), moderately morning type (score 59–69), intermediate type (score 42–58), moderately evening-type (score 31–41), and absolutely evening type (score 16–30) [34,35].

#### 2.4.6. Pittsburgh Sleep Quality Index (PSQI)

The Pittsburgh Sleep Quality Index (PSQI) is a self-rated questionnaire used to evaluate sleep quality over a 1-month time interval. It consists of seven main components (i.e., subjective sleep quality, sleep latency, sleep duration, habitual sleep efficiency, sleep disturbances, use of sleeping medication, and daytime dysfunction) for a total of 19 items. The composite PSQI score is the sum of seven component scores, and it indicates the overall sleep quality. In particular, a composite score lower or equal to 5 indicates good sleep quality, a score between 6 and 10 indicates poor sleep quality, whereas a score higher than 10 indicates very poor sleep quality [36,37].

#### 2.4.7. Epworth Sleepiness Scale (ESS)

The Epworth Sleepiness Scale (ESS) is a self-administered questionnaire measuring the general level of daytime sleepiness, and it is composed of 8 questions. Subjects are asked to rate, from 0 to 3, their usual likelihood of falling asleep during 8 diverse situations (with 0 meaning no chance of dozing; 1 meaning light chance of dozing; 2 meaning moderate chance of dozing; and 3 meaning high chance of dozing). The total score is the sum of the score obtained in each of the 8 items. A score higher than 10 suggests that the subject may suffer from daily sleepiness [37,38,39].

### 2.5. Ethical Issues

This study was carried out in accordance with the Declaration of Helsinki’s ethical standards. The study protocol was approved by the Ethical Committee of University Hospital “G. Martino”, Messina (C.E. prot. 40/19, dated 27 September 2019). Written informed consent was obtained from the participants to participate in the current investigation.

### 2.6. Statistical Analyses

Data processing and statistical analyses were performed using IBM SPSS Statistics v23 for Windows (IBM Corp, Armonk, NY, USA) and GraphPad Prism version 9.0 for Windows (GraphPad Software, La Jolla, CA, USA). For each variable of interest, Kolmogorov–Smirnov normality tests were run. The results (central tendencies and variance) are presented as frequencies for categorical variables and as mean value plus/minus standard deviation for continuous variables. Since all continuous variables follow a non-normal distribution, non-parametric tests were used, and the Mann–Whitney U test was used to assess differences between two groups. Spearman correlation analyses with determination of Spearman correlation r coefficients were conducted to evaluate occurring associations among diverse salivary biomarker levels and among different questionnaires. Contingency analyses were performed using Chi-squared (χ^2^) tests to evaluate occurring relationships between salivary biomarker levels and questionnaires outcomes. Biomarker data were dichotomized according to limit range values for each analyzed salivary biomarker (i.e., high and low morning cortisol, high and low evening cortisol, high and low alpha-amylase, and high and low melatonin). Limit range values were determined according to salivary kit manufacturer’s indications (see Supplementary Table S1). Accordingly, questionnaires data were divided into BDI > 9 (presence of depressive symptoms) and BDI ≤ 9 (absence of depressive symptoms), HAM-A > 7 (presence of anxiety) and HAM-A ≤ 7 (absence of anxiety), E/R ratio ≥ 1 (presence of work-related stress) and E/R Ratio < 1 (absence of work-related stress), WAI ≤ 36 (poor/moderate work ability) and WAI > 36 (good/excellent work ability), PSQI > 5 (poor sleep quality) and PSQI ≤ 5 (good sleep quality), and ESS > 10 (presence of daytime sleepiness) and ESS ≤ 10 (absence of daytime sleepiness). For all statistical analyses, differences were considered significant with *p*-values < 0.05, with * *p* < 0.05; ** *p* < 0.01; *** *p* < 0.001; and **** *p* < 0.0001.

## 3. Results

### 3.1. Study Population Features and Observed Differences between Males and Females Nurses

A total of 102 nurses, 55 women and 47 men, participated in the study. Their sociodemographic characteristics, life habits, and work-related factors are reported in Table 1.

Upon stratification of the study population based on sex, no differences were observed regarding age (average 46 ± 11 years), marital status, and parental status. Conversely, men stayed in education one year longer than women. Regarding health status parameters, while heart rate, and systolic and diastolic blood pressure were not statistically different between males and females, men had a higher body mass index than women. Regarding lifestyle habits, about 30% of the entire population of nurses declared that they smoke tobacco cigarettes, although no significant differences were observed between males and females. Instead, regarding alcohol consumption, 43% of men declared consuming alcohol regularly, compared the 18% of women (*p* = 0.007). No difference was found among the two groups regarding work injuries and work seniority (average 19 ± 11 years).

### 3.2. Salivary Biomarker Detection and Level Correlation

Morning saliva samples were collected for all the 102 subjects, in which cortisol, alpha-amylase, and melatonin levels were analyzed. Evening saliva was analyzed only in 76 individuals (26 individuals out of 102 did not deliver their evening saliva samples). The measured concentrations of salivary biomarkers and the analysis of differences observed upon stratification into two groups based on gender are summarized below in Table 2.

According to the results obtained within the whole nurse population in this study, the measured average morning cortisol levels were 0.492 µg/dL (minimum detected value 0.001 µg/dL and maximum detected value 2.240 µg/dL), the evening cortisol levels were 0.146 µg/dL (minimum value 0.001 µg/dL and maximum value 3.150 µg/dL), the morning alpha-amylase levels were 125.3 U/mL (minimum value 2.3 U/mL and maximum value 555.0 U/mL), and the morning melatonin levels were 13.0 pg/mL (minimum value 0.01 pg/mL and maximum value 52.5 pg/mL). The average values of morning cortisol were significantly higher than those of evening cortisol, also following stratification according to sex type (Supplementary Figure S1). Additionally, upon stratification of the population based on gender type, no significant differences in levels between men and women were observed for any of the evaluated salivary biomarkers.

**Figure 2:**
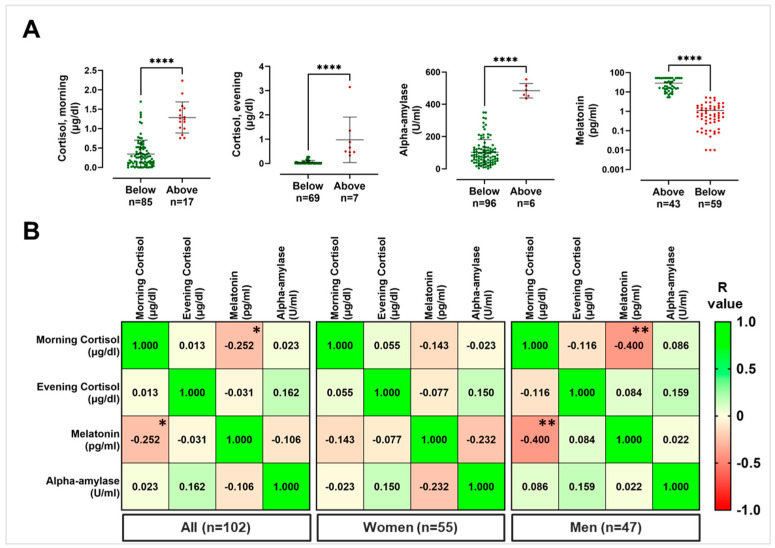
Salivary biomarkers analyses in nurses. (**A**) Salivary biomarker levels in nurse population; green dots indicate samples with values within the threshold limits, red dots indicate samples with values above (for cortisol and alpha-amylase) or below (for melatonin) the threshold value. (**B**) Matrix with correlation analyses among salivary biomarkers; in each square is indicated the *r* value, asterisks indicate a significant correlation, whereas no asterisk indicates absence of significant correlation. * *p* < 0.05; ** *p* < 0.01; **** *p* < 0.0001.

For what concerns morning cortisol and according to threshold levels normalized by the age, a total of 17 nurses out of 102 showed a measured value above the defined healthy cutoff, meaning that 17% of the nurses included in this study showed morning salivary cortisol values above the threshold limit. Regarding evening cortisol, 7 out of 76 subjects showed salivary levels above the threshold physiological limit, corresponding to 9% of the total. Concerning salivary alpha-amylase, 6 out of 102 nurses showed values above the threshold value (corresponding to the 6% of the total). Finally, for salivary melatonin, 59 out of 102 nurses, corresponding to the 58% of the population, showed values lower than the threshold value (Figure 2A). Notably, no significant differences were detected in terms of percentage of individuals above (in case of cortisol and alpha-amylase) or below (in case of melatonin) the physiological threshold value, when the population was stratified according to sex (Table 2).

Furthermore, in order to evaluate whether a correlation might occur between the detected salivary levels of cortisol (both morning and evening), alpha-amylase, and melatonin within the whole nurse population, correlation analyses were conducted. As shown in Figure 2B and summarized in Supplementary Table S2, when considering the whole nurse population, a negative correlation was observed between morning cortisol levels and morning melatonin levels (*r* value = −0.252, 95% CI = −0.430 to −0.054, *p* = 0.010). This negative correlation was also observed in both females and males, when considered separately, although it was statistically significant only within men group (*r* value = −0.400, CI = −0.622 to −0.120, *p* = 0.005). In particular, 12 out of 102 nurses (corresponding to 12% of the total) showed salivary morning cortisol and melatonin, respectively, above and below the threshold limit (Supplementary Figure S2).

### 3.3. Psychodiagnostics Questionnaires and Features of Nurse Population upon Stratification Based on Gender

BDI, HAM-A, ERI, and WAI might be considered complementary indexes of general work-related stress. Hence, higher scores of BDI, HAM-A, and ERI and lower scores of WAI are associated with higher stress perceived by nurses at the workplace, and vice versa.

The analysis of the results obtained for stress-related questionnaires within the entire sample in study, as well as upon stratification into the female and male subgroups, is showed in Table 3.

The BDI results revealed an overall low prevalence of depressive symptoms among all nurses. In detail, 86.3% were ranked as normal, corresponding to the absence of depressive symptoms, whereas 10.8% were scored as having minimal depressive symptoms and 2.9% were scored as having mild depressive symptoms. When the nurse population was stratified based on sex, a significantly different distribution of relative percentage frequencies was shown between women and men, respectively, with 78.2% and 95.8% ranking as normal, 18.2% and 2.1% scoring as having minimal symptoms, and 3.6% and 2.1% scoring as having mild symptoms (*p* = 0.028).

The HAM-A results revealed an overall low prevalence of anxiety symptoms among all nurses. In detail, 72.5% scored as normal, corresponding to the absence of anxiety symptoms, whereas 13.7% scored as having mild anxiety symptoms, 7.8% scored as having moderate anxiety, 2.9% scored as having severe anxiety, and 2.9% scored as having extremely severe anxiety. When the nurse population was stratified based on sex, a significantly different distribution of relative percentage frequencies was shown between women and men, respectively, with 60.0% and 87.2% ranking as normal, 20.0% and 6.4% scoring was shown mild symptoms, 12.7% and 2.1% scoring was shown moderate symptoms, and 3.6% and 2.1% scoring was shown both severe and extremely severe anxiety (*p* = 0.040).

**Figure 3:**
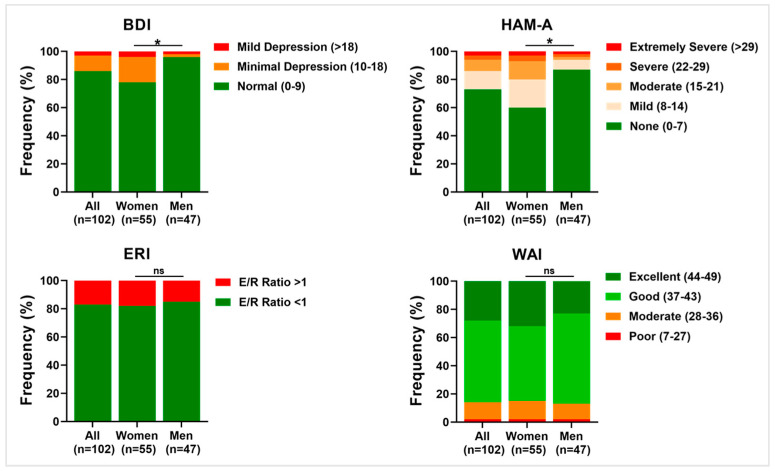
Distribution of relative percentage frequencies within nurses in stress-related questionnaires. The plots show the percentage distribution of the nurses for the whole population (left columns), as well as stratified into women (middle columns) and men (right columns) within the four scales relative to work-related stress: Beck Depression Inventory (BDI), Hamilton Anxiety Scale (HAM-A), Effort Reward Imbalance (ERI), and Work Ability Index (WAI). Green indicates positive outcomes, yellow and orange indicate intermediate outcomes, and red shows negative outcomes. * *p* < 0.05; ns, not significant.

Concerning both the ERI scale and the WAI scale, no differences were observed when stratifying the samples based on sex. ERI prevalence within the nurses was represented by an E/R ratio < 1, which indicates the prevalence of rewards compared with effort, compared with subjects scoring E/R ratio > 1, respectively 83.3% versus 16.7%. The WAI questionnaire highlighted the prevalence of good working abilities within the nurse population, compared with individuals falling into worse categories, 57.8% versus 2% scoring poor work ability and 11.8% scoring moderate work ability, and into a better category, 28.4% scoring excellent ability to work. The distribution of relative percentage frequencies of work-related stress indexes within the nurses is summarized below in Figure 3.

Among the psychodiagnostics questionnaires administered to the nurses, the MEQ assessed the chronotype, the PSQI scored the perceived quality of sleep, whereas the ESS assessed the degree of daytime sleepiness. All three questionnaires might be considered complementary indexes of how sleep quality may be affected by work activity. In particular, higher scores of PSQI and ESS are associated with worse sleep quality perceived by nurses at the workplace, and vice versa.

The analysis of the results obtained for sleep-related questionnaires within the entire sample in study, as well as upon stratification into the female and male subgroups, is shown in Table 4.

**Figure 4:**
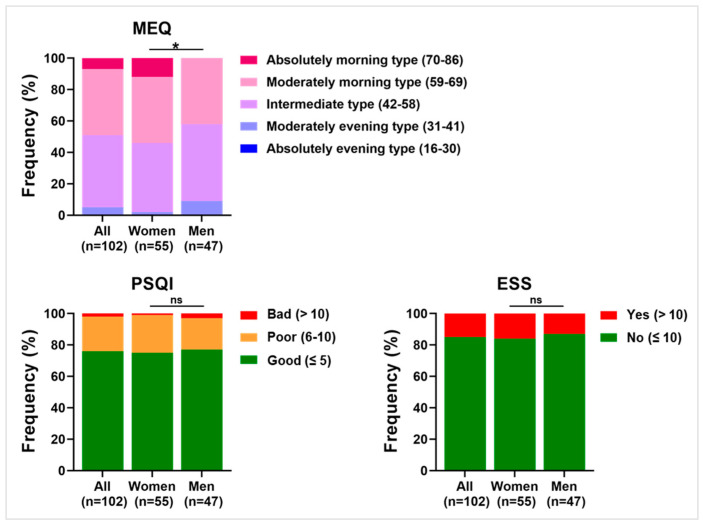
Distribution of relative percentage frequencies within nurses in sleep-related questionnaires. The plots show the percentage distribution of the nurses for the whole population (left columns), as well as when stratified into women (middle columns) and men (right columns) within the three scales relative to work-related sleep quality: Morning Evening Questionnaire (MEQ), Pittsburgh Sleep Quality Index (PSQI), and Epworth Sleepiness Scale (ESS). In MEQ, burgundy indicates morning type, the purple shades indicate intermediate types, and blue indicates evening types. In PSQI and ESS, green indicates positive outcomes, orange indicates intermediate outcomes, and red shows negative outcomes. * *p* < 0.05; ns, not significant.

Regarding the chronotype, the majority of the nurses were determined to be of either an intermediate type or moderately morning chronotype (respectively, 46.1% and 42.2%). Upon gender stratification, 12.7% of women were determined to be of an absolute morning type compared with 0.0% of men (*p* = 0.037). PSQI showed a prevalence of good outcome (75.5% of nurses) compared with poor and bad (respectively, 21.6% and 2.9%). No significant differences in the frequency distribution were observed when the sample was stratified based on sex type. Finally, ESS highlighted that the majority of subjects experienced no daytime sleepiness (85.3% of the total). This proportion is maintained also when the nurse population is divided into females and males. Figure 4 summarizes the distribution of relative percentage frequencies of work-related sleep quality indexes within the nurses.

### 3.4. Psychodiagnostics Questionnaire Correlation Study

**Figure 5:**
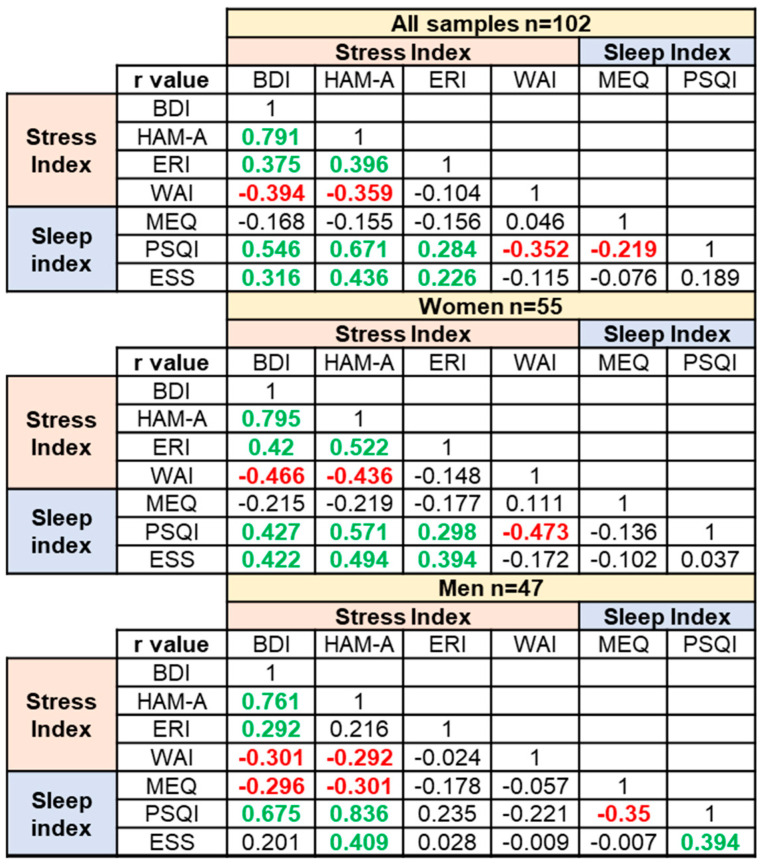
Correlation analyses among all psychodiagnostics questionnaires. Correlation matrix including all samples (top panel), women (middle panel), and men (bottom panel). Four scales relative to work-related stress: Beck Depression Inventory (BDI), Hamilton Anxiety Scale (HAM-A), Effort Reward Imbalance (ERI), and Work Ability Index (WAI). Three scales relative to work-related sleep quality: Morning Evening Questionnaire (MEQ), Pittsburgh Sleep Quality Index (PSQI), and Epworth Sleepiness Scale (ESS). Green *r* values indicate positive correlation, and red *r* values indicate negative correlation.

Upon linear transformation of the questionnaire scores, for each questionnaire, the common scale had a minimum of 0 and a maximum of 1, and a Spearman correlation study between the outcomes obtained for all questionnaires was performed. The correlation study was conducted for the whole population of nurses, as well as after stratification into the female and male nurses. The results are shown in Figure 5, while the corresponding *p*-values and confidence intervals are summarized in Supplementary Table S3.

In the whole nurse population and considering the work-related stress questionnaires, a significantly positive correlation was found between the BDI and HAM-A scales, between the BDI and ERI scales, and between the HAM-A and ERI scales, whereas WAI negatively and significantly correlated with both BDI and HAM-A (negatively but not significantly correlated with ERI). The overall correlations were maintained also when the samples are stratified based on sex type.

In the whole nurse population and considering the work-related sleep questionnaires, a significant negative correlation between MEQ and PSQI was observed, which is significant in the male group, but not in the female one. Only in men was a significantly positive correlation observed between PSQI and ESS outcomes.

Considering the whole nurse population, the correlation between stress questionnaires outcomes and sleep questionnaires outcomes was analyzed. A significantly positive correlation of PSQI with BDI, HAM-A, and ERI and a significantly negative correlation with WAI were observed. Although the trend was maintained, the significance of PSQI correlations, both with ERI and WAI, was maintained in women, but not in men, following stratification. ESS was significantly and positively correlated with the BDI, HAM-A, and ERI outcomes in the whole population, although the significance between ESS and ERI correlation was lost in the men group upon stratification.

### 3.5. Contingency Studies Highlighted a Tight Link between Salivary Morning Cortisol Levels and Depressive Symptoms and between Salivary Morning Alpha-Amylase and Work Ability

In order to assess the relationships between measured salivary biomarker levels and psychodiagnostics questionnaires, a contingency analysis with χ^2^ tests was conducted. Table 5 is a summary of the results of the contingency analyses between work-related stress scales and salivary biomarkers. The frequencies relative to the contingency analyses between work-related stress scales and salivary biomarkers are summarized in Supplementary Table S4.

**Figure 6:**
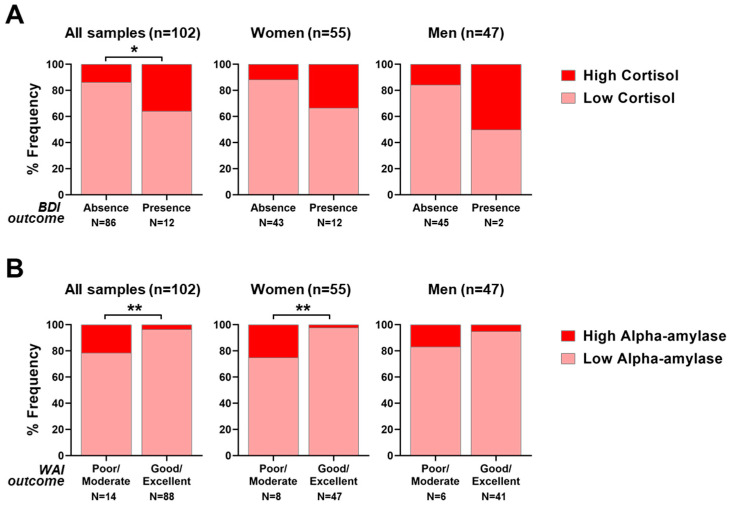
Contingency analyses of psychodiagnostics questionnaires and salivary biomarkers. (**A**) Beck Depression Inventory (BDI) outcome (absence versus presence of depressive symptoms, respectively, left and right columns) in individuals with high (red) versus low (pink) cortisol; considering all samples (left plot), female group (middle plot), male group (right plot). (**B**) Work Ability Index (WAI) outcome (poor or moderate work ability versus good or excellent work ability, respectively, left and right columns) in individuals with high (red) versus low (pink) alpha-amylase; considering all samples (left plot), female group (middle plot), male group (right plot). * *p* < 0.05; ** *p* < 0.01.

Such an analysis has been carried out within the whole population, as well as following stratification into the women and men subgroups. The contingency analysis of salivary cortisol (both morning and evening), alpha-amylase (morning), melatonin (morning) with work-related stress questionnaires evidenced a significantly positive interdependence between morning cortisol levels and BDI, which is the depression scale, with a χ^2^ value of 4.24 (*p* = 0.040, Figure 6A). However, the significance is lost when the samples are stratified according to sex type. A significantly positive relationship was also observed between salivary alpha-amylase levels and WAI outcome, which reflects the work ability perception, with a χ^2^ value of 7.08 (*p* = 0.008, Figure 6B) considering all samples and a χ^2^ value of 6.94 (*p* = 0.008) considering only the female group. Such significance is lost when considering only male nurses.

Additionally, the occurrence of significant relationships was analyzed between work-related perceived sleep quality scales and salivary biomarkers levels within the whole nurse population, as well as following stratification into males or females. The result showed a positive relationship between salivary morning cortisol and sleepiness (ESS scale) when considering all subjects, although not significant (χ^2^ = 3.52, *p* = 0.061). All results obtained are reported below in Table 6. The frequencies relative to contingency analyses between perceived sleep quality scales and salivary biomarkers are summarized in Supplementary Table S5.

## 4. Discussion

A total of 102 nurses, 55 women (54%) and 47 men (46%), voluntarily participated in this exploratory study. On the date of assessment, nurses’ ages ranged between 23 and 68 years, with working seniority being between 1 year and 37 years. Salivary biomarkers were analyzed in order to identify how many workers had levels that could be indicative of either stress (i.e., salivary cortisol and alpha-amylase levels above the healthy threshold) or sleep disorders (i.e., salivary melatonin below the healthy threshold level) [16,40,41]. Overall, considering the whole nurse population, 17 out of 102 showed morning cortisol above the threshold limit (corresponding to 17% of the total). However, when stratified based on the age, the morning cortisol levels were differently distributed. In particular, within the youngest individuals (age 21–30), 41.7% (corresponding to 5 individuals out of 12) had morning cortisol levels above the threshold. Despite the results needing further validation, these data are in line with what is observed by others, highlighting that shift work might trigger a morning cortisol surge in the youngest subjects [42]. Indeed, as already observed in our previous study conducted in a similar sample of hospital workers, old age can be considered protective against stress; hence, a shorter work history may lead to maladaptive stress-coping strategies [43,44]. Importantly, a morning cortisol increase in the youngest individuals has been positively associated with augmented cardiovascular risk, anxiety, chronic stress, and depression [45,46,47].

Concerning evening salivary cortisol and morning alpha-amylase, respectively, 91% and 94% of nurses had values below the threshold limit. Both percentages were maintained also when samples where stratified into females and males. These findings suggest that the adopted clockwise rotation shift effectively helps nurses to positively adapt to night shift, hence protecting the majority of the workers from detrimental spikes in both cortisol or alpha-amylase production. This observation is in line with previous ones, both in healthcare workers and in other occupational settings [24,48,49].

Diversely, 58% of the nurses showed morning melatonin salivary levels below the threshold value, therefore considered non-physiological. This percentage did not significantly differ when samples were stratified by sex type. The observed result may suggest that, among the salivary biomarkers analyzed, melatonin represents a powerful early indicator of circadian clock alteration, and it is the most influenced by night shift work in our sample. Indeed, compared with morning work, night shift work can be associated with up to a 34% reduction in morning melatonin production [50]. However, in our sample, as suggested by contingency analyses, salivary melatonin levels are not in a significant relationship with any of the sleep questionnaire outcomes, suggesting an overall sleep wellbeing and positive adaptation to clockwise rotation shifts among nurses. In future studies, in order to support subjective awareness, the concurrent utilization of wearable devices would be useful to instrumentally assess the actual sleep patterns [51].

Considering all nurses, a negative correlation was also observed between salivary morning cortisol levels and morning melatonin levels. These reported observations are in line with existing scientific evidence [52]. Indeed, as shown in Supplementary Figure S2, within the nurse population, a small subgroup of 12 subjects (12% of the total) with high cortisol and low melatonin who might be considered at higher potential risk of developing stress and/or sleep disturbances could be identified.

For what concerns psychodiagnostics questionnaires, all examined direct and indirect stress indexes highlighted that a small percentage of the nurses experienced work-related stress (ranging between 13% and 17%, depending on the test). However, when nurses were stratified based on sex, a higher percentage of females reported mild depressive and anxious symptoms compared to men (i.e., 78.2% versus 95.8% and 60.0% versus 87.2% ranked normal, respectively, on the BDI and HAM-A scales). This result is in line with several studies showing that females, more than males, might be more self-aware of work-related stress [53,54,55]. Additionally, this finding was further supported by evidence within the different occupational settings, where it was demonstrated that females have almost twice the risk of developing depression, anxiety, and other stress-related disorders upon circadian disruption [56]. From this perspective, workplace health promotion programs should be implemented to support and enhance balance and wellbeing, especially among more vulnerable female employees [57,58].

The sleep-related questionnaires showed that 76% of nurses consider their sleep quality to be good. Additionally, 85% of the study population declared the absence of sleepiness during daytime. Both results were comparable when the sample was stratified based on gender type. Conversely, it was possible to observe chronotype differences. While the whole population scored intermediate and moderate morning chronotypes, when the samples were stratified, females showed a higher percentage of absolute morning types compared with males (respectively, 13% compared with none). This observation further strengthens the idea that men are more evening oriented than women [59]. Importantly, a very early chronotype tends to poorly adapt to working night shifts, and this might explain the observed increased prevalence of mild depression and anxiety symptoms in women [60].

Interestingly, the questionnaires outcomes showed a significant correlation between each other (Figure 5). Although answering multiple surveys can be a time-consuming task for the workers, it allows for the results from different scales to be matched to obtain a broader perspective of a given condition. Indeed, we considered multiple indicators directly or indirectly correlated with work-related stress, as well as sleep wellness. Despite the possible burden of answering many questionnaires, the significant correlations obtained between diverse questionnaires’ outcomes demonstrated that nurses responded with coherence. Additionally, these data are suggestive of a reliable degree of self-awareness of the interviewed subjects. Overall, these correlations corroborate the importance of a multi-assessment approach to measure self-perception of potential stress and sleep imbalance in nurses working shift schedules, including night shifts [61].

Finally, this study evaluated whether it might subsist any interdependence between the objective biological readouts from salivary biomarkers and the subjective outcomes from the self-administered questionnaires. Interestingly, two significant relationships have been found. Firstly, subjects who scored higher on the BDI questionnaire (declaring a perception of stronger depressive feelings) also had a higher level of morning cortisol in their saliva (which is an index of chronic stress), and vice versa. Secondly, nurses who scored higher on the WAI questionnaire (declaring good to excellent perception of ability to work) also had a low level of morning salivary alpha-amylase (which is an index of acute stress), and vice versa.

While the relationship between cortisol and depression has been studied and some groups found correlations between depression scales and serum cortisol, the interdependence between alpha-amylase and work ability is new, and it deserves to be further characterized in the future [62,63]. In our previous study, conducted in male dock night shift workers, we found a negative relationship between alpha-amylase and the effort/reward imbalance score [19]. Although this finding corroborates the reliability of alpha-amylase as an early indicator of work-related stress, the differences between the outcomes in the two studies might be due to the very diverse occupational backgrounds examined. Firstly, there is a difference in gender proportion. While dock workers were males, nurses are a mixed population. Secondly, the work tasks are different. While dock workers carried out their jobs outdoors, facing potentially straining and dangerous conditions, which might require strong physical engagement, nurse tasks are carried out indoors, with tasks that are prevalently mentally demanding. As suggested by the results obtained, in the presence of heterogeneous stress triggers (which can alter salivary alpha-amylase secretion), nurses may be more aware of work ability imbalance, while dock workers may perceive an imbalance in the effort compared with the reward.

Generally, for workers employed in the healthcare system, especially nurses, shift work and night shift work represent occupational hazards that expose workers to higher risks of developing both sleep disorders and stress symptomatology. This may bring on increased sick leave, burnout, and more frequent occupational accidents and clinical errors [64]. Overall, this pilot study demonstrates that during mandatory medical surveillance, the additional use of a multi-assessment approach, with an analysis of selected salivary biomarkers, as well as a survey through a battery of complementary psychodiagnostics questionnaires, might help the occupational physician to better identify personnel at higher risk of developing work-related stress and/or sleep disturbances.

This exploratory analysis represents a proof-of-concept study which deserves to be further validated in larger populations. From this perspective, in the future, it would be useful to further extend the salivary biomarker analyses to different timepoints, given the circadian pattern of their expression. Additionally, it could be interesting to evaluate additional biomarkers that could be correlated either with stress or with sleep quality (e.g., immunoglobulin A, lysozyme, and chromogranin A). Regarding the questionnaires, it would be helpful to assess potential coping strategies that shift workers might use in response to stress. In this direction, sociodemographic profiles have been outstanding in human health research, and now, the perception of social support has been linked with inflammatory response, emotional functioning, and several health indices [65,66]. Hence, it would be relevant to additionally explore these aspects in correlation with salivary wellbeing indexes in the near future.

## 5. Conclusions

This pilot study assessed both objective (salivary biomarkers) and subjective (psychodiagnostics questionnaires) parameters in a population of nurses working shift schedules, including night shifts. In particular, the biological monitoring of salivary cortisol, alpha-amylase, and melatonin was associated with the administration of several psychodiagnostics questionnaires assessing either stress-related parameters or sleep habits and quality. The use of multiple questionnaires allowed us to assess and correlate different aspects of work-related stress and sleep disorders, which might potentially increase their incidence in shift workers, especially in nurses often subjected to heavy workloads. Through the analyses hereby performed, it was possible to establish significant relationships between detected biomarkers levels and specific questionnaire outcomes, highlighting the importance of a multi-assessment approach to facilitate medical surveillance programs and to implement novel health preventive strategies. Importantly, this study is limited to an exploratory sample of 102 males and female nurses. To strengthen such conclusions, in the future, it will be pivotal to further validate the results in larger study groups.

## Figures and Tables

**Table 1:** Description of study population, differences upon stratification into male and female subgroups.

	Total	Women	Men	*p*-Value
	*n* (%)	*n* (%)	*n* (%)	
Total	102 (100)	55 (53.9)	47 (46.1)	
Age				
Mean ± SD	46.17 ± 11.02	47.60 ± 11.40	44.49 ± 10.43	0.150
Education (years)				
Mean ± SD	13.75 ± 1.93	13.27 ± 2.13	14.32 ± 1.49	**0.012**
Marital status				
Single	23 (22.5)	10 (18.2)	13 (27.7)	0.254
Married/Cohabitant	79 (77.5)	45 (81.8)	34 (72.3)	
Parental status				
No	33 (32.4)	15 (27.3)	18 (38.3)	0.235
Yes	59 (67.6)	40 (72.7)	29 (61.7)	
Body Mass Index				
Mean ± SD	26.86 ± 4.98	25.82 ± 4.68	28.08 ± 5.09	**0.023**
Systolic Blood Pressure				
Mean ± SD	118.91 ± 17.94	116.89 ± 21.35	121.28 ± 12.70	0.428
Diastolic Blood Pressure				
Mean ± SD	76.83 ± 10.60	76.40 ± 12.01	77.34 ± 8.78	0.520
Heart Rate				
Mean ± SD	75.21 ± 12.82	77.00 ± 13.29	73.11 ± 12.04	0.138
Smoking habit				
No	71 (69.6)	42 (76.4)	29 (61.7)	0.109
Yes	31 (30.4)	13 (23.6)	18 (38.3)	
Alcohol consumption				
No	72 (70.6)	45 (81.8)	27 (57.4)	**0.007**
Yes	30 (29.4)	10 (18.2)	20 (42.6)	
Medications				
No	55 (53.9)	25 (45.5)	30 (63.8)	0.063
Yes	47 (46.1)	30 (54.5)	17 (36.2)	
Work injury				
No	85 (83.3)	46 (83.6)	39 (83.0)	0.929
Yes	17 (16.7)	9 (16.4)	8 (17.0)	
Seniority (years)				
Mean ± SD	18.72 ± 10.80	19.71 ± 11.31	17.55 ± 10.16	0.317

Abbreviations: SD, standard deviation. Bold text indicates significant differences.

**Table 2:** Levels of measured salivary biomarkers, differences upon stratification in male and female subgroups.

	Total	Women	Men	*p*-Value
	*n* (%)	*n* (%)	*n* (%)	
Morning cortisol (µg/dL) (*n* = 102)				
Mean ± SD	0.492 ± 0.500	0.438 ± 0.428	0.556 ± 0.571	0.252
Morning cortisol (µg/dL) in Age 21–30 (*n* = 12) ^1^				
<0.743 *	7/12 (58.3)	3/6 (50.0)	4/6 (66.7)	0.999
>0.743	**5**/12 (41.7)	3/6 (50.0)	2/6 (33.3)	
Morning cortisol (µg/dL) in Age 31–50 (*n* = 52) ^2^				
<1.551 *	49/52 (94.2)	24/24 (100.0)	25/28 (89.3)	0.240
>1.551	**3**/52 (5.8)	0/24 (0.0)	3/28 (10.7)	
Morning cortisol (µg/dL) in Age 51–70 (*n* = 38) ^3^				
<0.812 *	29/38 (76.3)	19/25 (76.0)	10/13 (76.9)	0.999
>0.812	**9**/38 (23.7)	6/25 (24.0)	3/13 (23.1)	
Evening cortisol (µg/dL) (*n* = 76)				
Mean ± SD	0.146 ± 0.411	0.159 ± 0.487	0.129 ± 0.289	0.996
Evening cortisol (µg/dL) in Age 21–30 (*n* = 10) ^1^				
<0.308 *	8/10 (80.0)	4/5 (80.0)	4/5 (80.0)	0.999
>0.308	**2**/10 (20.0)	1/5 (20.0)	1/5 (20.0)	
Evening cortisol (µg/dL) in Age 31–50 (*n* = 41) ^2^				
<0.359 *	37/41 (90.2)	20/21 (95.2)	17/20 (85.0)	0.343
>0.359	**4**/41 (9.8)	1/21 (4.8)	3/20 (15.5)	
Evening cortisol (µg/dL) in Age 51–70 (*n* = 25) ^3^				
<0.228 *	24/25 (96.0)	17/17 (100.0)	7/8 (87.5)	0.320
>0.228	**1**/25 (4.0)	0/17 (0.0)	1/8 (12.5)	
Alpha-Amylase (U/mL) (*n* = 102)				
Mean ± SD	125.3 ± 118.9	124.3 ± 116.2	126.5 ± 123.4	0.989
Alpha-Amylase Range (U/mL)				
<423.1 *	96 (94.1)	52 (94.5)	44 (93.6)	0.540
>423.1	**6** (5.9)	3 (5.5)	3 (6.4)	
Melatonin (pg/mL) (*n* = 102)				
Mean ± SD	13.0 ± 18.2	15.2 ± 19.6	10.4 ± 16.3	0.224
Melatonin Range (pg/mL)				
<5.2	**59** (57.8)	28 (50.9)	31 (66.0)	0.125
>5.2 *	43 (42.2)	27 (49.1)	16 (34.0)	

Abbreviations: SD, standard deviation; * limit range values; ^1^ Percentages calculated on the number of participants with Age 21–30; ^2^ Percentages calculated on the number of participants with Age 31–50; ^3^ Percentages calculated on the number of participants with Age 51–70. Bold text indicates frequency of samples above or below the threshold limit value.

**Table 3:** Psychodiagnostics protocol results concerning overall work-related stress evaluation.

	Total	Women	Men	*p*-Value
	*n* (%)	*n* (%)	*n* (%)	
Beck Depression Inventory (BDI)				
Mean ± SD	4.65 ± 5.22	5.65 ± 5.67	3.47 ± 4.39	**0.020**
Normal (0–9)	88 (86.3)	43 (78.2)	45 (95.8)	**0.028**
Minimal Depression (10–18)	11 (10.8)	10 (18.2)	1 (2.1)	
Mild Depression (>18)	3 (2.9)	2 (3.6)	1 (2.1)	
Hamilton Anxiety Scale (HAM–A)				
Mean ± SD	6.11 ± 7.22	7.64 ± 7.45	4.32 ± 6.56	**0.003**
None (0–7)	74 (72.5)	33 (60.0)	41 (87.2)	**0.040**
Mild (8–14)	14 (13.7)	11 (20.0)	3 (6.4)	
Moderate (15–21)	8 (7.8)	7 (12.7)	1 (2.1)	
Severe (22–29)	3 (2.9)	2 (3.6)	1 (2.1)	
Extremely Severe (>29)	3 (2.9)	2 (3.6)	1 (2.1)	
Effort Reward Imbalance (ERI)				
Effort (E)	7.48 ± 2.71	7.64 ± 2.82	7.30 ± 2.58	0.277
Reward (R)	20.24 ± 3.43	20.49 ± 3.39	19.94 ± 3.49	0.923
Overcommitment (O)	11.78 ± 3.62	12.36 ± 3.95	11.11 ± 3.10	0.125
Effort/Reward Ratio (E/R)	0.82 ± 0.18	0.823 ± 0.179	0.817 ± 0.186	0.906
E/R Ratio < 1	85 (83.3)	45 (81.8)	40 (85.1)	0.657
E/R Ratio > 1	17 (16.7)	10 (18.2)	7 (14.9)	
Work Ability Index (WAI)				
Mean ± SD	40.69 ± 5.12	40.85 ± 4.91	40.51 ± 5.39	0.620
Poor (7–27)	2 (2.0)	1 (1.8)	1 (2.1)	0.701
Moderate (28–36)	12 (11.8)	7 (12.7)	5 (10.6)	
Good (37–43)	59 (57.8)	29 (52.7)	30 (63.8)	
Excellent (44–49)	29 (28.4)	18 (32.7)	11 (23.4)	

Abbreviations: SD, standard deviation. Bold text indicates significant differences.

**Table 4:** Psychodiagnostics protocol results concerning overall sleep quality evaluation.

	Total	Women	Men	*p*-Value
	*n* (%)	*n* (%)	*n* (%)	
Morning Evening Questionnaire (MEQ)				
Mean ± SD	58.10 ± 8.13	62.99 ± 11.54	56.81 ± 10.91	**0.010**
Absolutely evening type (16–30)	0 (0.0)	0 (0.0)	0 (0.0)	**0.037**
Moderately evening type (31–41)	5 (4.9)	1 (1.8)	4 (8.5)	
Intermediate type (42–58)	47 (46.1)	24 (43.6)	23 (48.9)	
Moderately morning type (59–69)	43 (42.2)	23 (41.8)	20 (42.6)	
Absolutely morning type (70–86)	7 (6.9)	7 (12.7)	0 (0.0)	
Pittsburgh Sleep Quality Index (PSQI)				
Mean ± SD	4.15 ± 2.84	4.33 ± 2.83	3.94 ± 2.88	0.467
Good (≤5)	77 (75.5)	41 (74.5)	36 (76.6)	0.683
Poor (6–10)	22 (21.6)	13 (23.6)	9 (19.1)	
Bad (>10)	3 (2.9)	1 (1.8)	2 (4.3)	
Epworth Sleepiness Scale (ESS)				
Mean ± SD	4.84 ± 5.27	4.38 ± 3.44	5.38 ± 6.82	0.753
No (≤10)	87 (85.3)	46 (83.6)	41 (87.2)	0.609
Yes (>10)	15 (14.7)	9 (16.4)	6 (12.8)	

Abbreviations: SD, standard deviation. Bold text indicates significant differences.

**Table 5:** Contingency analyses between work-related stress scales and salivary biomarkers.

	BDI		HAM-A		E/R Ratio		WAI	
	χ^2^	*p*-Value	χ^2^	*p*-Value	χ^2^	*p*-Value	χ^2^	*p*-Value
Morning cortisol								
Total	**4.24**	**0.040**	0.63	0.427	0.35	0.552	0.27	0.607
Women	3.23	0.072	1.09	0.298	0.36	0.548	0.51	0.475
Men	1.61	0.205	0.01	0.980	0.04	0.835	0.01	0.980
Evening cortisol								
Total	1.74	0.187	3.78	0.052	1.90	0.169	1.74	0.187
Women	0.81	0.368	2.00	0.157	0.64	0.425	0.48	0.489
Men	0.38	0.538	0.91	0.367	1.05	0.305	1.31	0.252
Alpha-amylase								
Total	0.05	0.829	0.11	0.739	1.28	0.259	**7.08**	**0.008**
Women	0.25	0.619	0.94	0.332	0.71	0.401	**6.94**	**0.008**
Men	0.14	0.706	0.47	0.493	0.56	0.454	1.22	0.270
Melatonin								
Total	0.01	0.954	0.29	0.591	0.01	0.929	1.23	0.268
Women	0.34	0.561	0.19	0.660	0.40	0.525	2.17	0.140
Men	0.24	0.626	0.78	0.377	0.29	0.594	0.01	0.969

Abbreviations: BDI, Beck Depression Inventory; HAM-A, Hamilton Anxiety Scale; ERI, Effort Reward Imbalance; WAI, Work Ability Index. Bold text indicates significant differences.

**Table 6:** Contingency analyses between perceived sleep quality scales and salivary biomarkers.

	PSQI		ESS	
	χ^2^	*p*-Value	χ^2^	*p*-Value
Morning cortisol				
Total	0.27	0.607	3.52	0.061
Women	0.35	0.553	2.26	0.132
Men	0.01	0.907	1.30	0.255
Evening cortisol				
Total	0.38	0.539	0.01	0.909
Women	0.72	0.396	0.48	0.489
Men	0.01	0.943	0.34	0.558
Alpha-amylase				
Total	0.21	0.645	1.10	0.294
Women	0.10	0.747	0.62	0.431
Men	0.98	0.322	0.47	0.493
Melatonin				
Total	0.46	0.496	0.56	0.454
Women	0.01	0.937	1.07	0.301
Men	0.83	0.361	0.01	0.969

Abbreviations: PSQI, Pittsburgh Sleep Quality Index; ESS, Epworth Sleepiness Scale.

## Data Availability

The datasets generated and analyzed during the current study are available from the corresponding author upon reasonable request.

## Supplementary Materials

The following supporting information can be downloaded at https://www.mdpi.com/article/10.3390/ijerph20075376/s1, Table S1: Limit range values of salivary biomarkers; Figure S1: Differences between levels of morning and evening salivary cortisol in a nurse population; Table S2: Correlation analyses among salivary biomarkers; Figure S2: Correlation dot plot between salivary melatonin and cortisol; Table S3: Correlation analyses among all psychodiagnostics questionnaires; Table S4: Frequencies relative to contingency analyses between work-related stress scales and salivary biomarkers; Table S5: Frequencies relative to contingency analyses between perceived sleep quality scales and salivary biomarkers.
