# Supplementary material for: Salivary Biomarkers Analysis and Neurobehavioral Assessment in Nurses Working Rotation Shifts: A Pilot Study
Source: Int J Environ Res Public Health. 2023 Apr 3;20(7):5376. doi: 10.3390/ijerph20075376 (PMC10094107; doi:10.3390/ijerph20075376)
Supplement: Supplementary file 1 [file ijerph-20-05376-s001.zip › ijerph-2187711-supplementary.pdf]

Supplementary Material

Salivary Biomarkers Analysis and Neurobehavioral Assessment in Nurses Working Rotation Shifts: A Pilot Study

Silvia Vivarelli [1,†], Sebastiano Italia [1,†], Michele Teodoro [1], Manuela Pollicino [1], Carmen Vitello [1], Annalisa De Vita [1], Angela Alibrandi [2], Chiara Costa [3,*] and Concettina Fenga [1,*]

[1] Department of Biomedical and Dental Sciences, Morphological and Functional Imaging, Section of Occupational Medicine, University of Messina, 98125 Messina, Italy
[2] Department of Economics, University of Messina, 98125 Messina, Italy
[3] Department of Clinical and Experimental Medicine, University of Messina, 98125 Messina, Italy
* Correspondence: chiara.costa@unime.it (C.C.); concettina.fenga@unime.it (C.F.)
† These authors contributed equally to this work.

Supplementary Table S1. Limit range values of salivary biomarkers.

Morning cortisol (µg/dl) in Age 21 – 30	
Low	< 0.743*
High	> 0.743
Morning cortisol (µg/dl) in Age 31 – 50	
Low	< 1.551*
High	>1.551
Morning cortisol (µg/dl) in Age 51 – 70	
Low	< 0.812*
High	> 0.812
Evening cortisol (µg/dl) in Age 21 – 30	
Low	< 0.308*
High	> 0.308
Evening cortisol (µg/dl) in Age 31 – 50	
Low	< 0.359*
High	> 0.359
Evening cortisol (µg/dl) in Age 51 – 70	
Low	< 0.228*
High	> 0.228
Alpha-Amylase (U/ml)	
Low	< 423.1*
High	> 423.1
Melatonin (pg/ml)	
Low	< 5.2
High	> 5.2 *

*Limit range values

Supplementary Figure S1. Differences between levels of morning and evening salivary cortisol in nurse population. **A.** Nurses (n = 76); **B.** Females (n = 43); **C.** Males (n = 33). **** $p < 0.0001$.

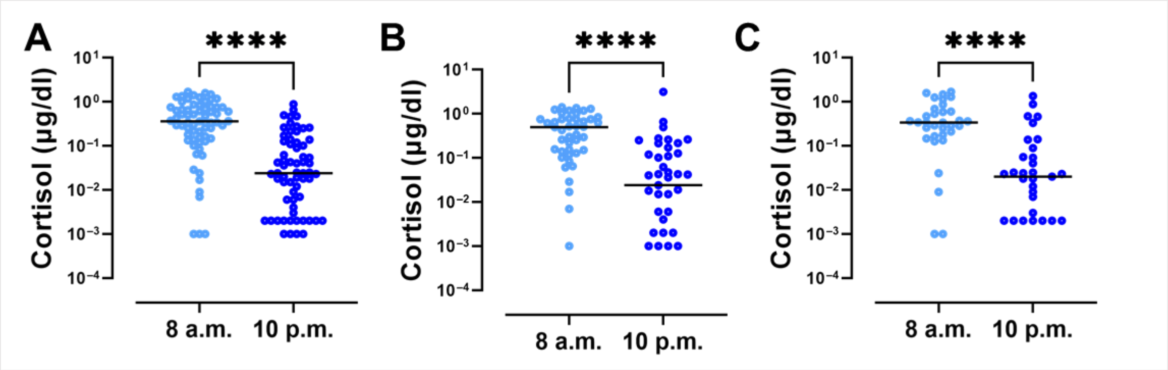

Supplementary Table S2. Correlation analyses among salivary biomarkers.

All samples				
<u>**Spearman r**</u>	**Morning Cortisol (µg/dl)**	**Evening Cortisol (µg/dl)**	**Melatonin (pg/ml)**	**Alpha-amylase (U/ml)**
**Morning Cortisol (µg/dl)**	1	0.013060376	**-0.251741301**	0.023476183
**Evening Cortisol (µg/dl)**	0.013060376	1	-0.031174419	0.162361883
**Melatonin (pg/ml)**	-0.251741301	-0.031174419	1	-0.105659347
**Alpha-amylase (U/ml)**	0.023476183	0.162361883	-0.105659347	1
<u>**95% CI**</u>	**Morning Cortisol (µg/dl)**	**Evening Cortisol (µg/dl)**	**Melatonin (pg/ml)**	**Alpha-amylase (U/ml)**
**Morning Cortisol (µg/dl)**	1	-0.2195 to 0.2442	**-0.4301 to -0.05441**	-0.1774 to 0.2225
**Evening Cortisol (µg/dl)**	-0.2195 to 0.2442	1	-0.2612 to 0.2022	-0.07224 to 0.3799
**Melatonin (pg/ml)**	-0.4301 to -0.05441	-0.2612 to 0.2022	1	-0.2994 to 0.09645
**Alpha-amylase (U/ml)**	-0.1774 to 0.2225	-0.07224 to 0.3799	-0.2994 to 0.09645	1
<u>**P values**</u>	**Morning Cortisol (µg/dl)**	**Evening Cortisol (µg/dl)**	**Melatonin (pg/ml)**	**Alpha-amylase (U/ml)**
**Morning Cortisol (µg/dl)**		0.910842851	**0.010699156**	0.814823524
**Evening Cortisol (µg/dl)**	0.910842851		0.789212704	0.161125313
**Melatonin (pg/ml)**	0.010699156	0.789212704		0.290549335
**Alpha-amylase (U/ml)**	0.814823524	0.161125313	0.290549335	

Women				
<u>**Spearman r**</u>	**Morning Cortisol (µg/dl)**	**Evening Cortisol (µg/dl)**	**Melatonin (pg/ml)**	**Alpha-amylase (U/ml)**
**Morning Cortisol (µg/dl)**	1	0.054630323	-0.143299152	-0.022826932
**Evening Cortisol (µg/dl)**	0.054630323	1	-0.076675027	0.149737413
**Melatonin (pg/ml)**	-0.143299152	-0.076675027	1	-0.23204914
**Alpha-amylase (U/ml)**	-0.022826932	0.149737413	-0.23204914	1

95% CI	Morning Cortisol (µg/dl)	Evening Cortisol (µg/dl)	Melatonin (pg/ml)	Alpha-amylase (U/ml)
**Morning Cortisol (µg/dl)**	1	-0.2584 to 0.3573	-0.4004 to 0.1347	-0.2937 to 0.2515
**Evening Cortisol (µg/dl)**	-0.2584 to 0.3573	1	-0.3764 to 0.2376	-0.1666 to 0.4381
**Melatonin (pg/ml)**	-0.4004 to 0.1347	-0.3764 to 0.2376	1	-0.4748 to 0.04345
**Alpha-amylase (U/ml)**	-0.2937 to 0.2515	-0.1666 to 0.4381	-0.4748 to 0.04345	1

P values	Morning Cortisol (µg/dl)	Evening Cortisol (µg/dl)	Melatonin (pg/ml)	Alpha-amylase (U/ml)
**Morning Cortisol (µg/dl)**		0.727886104	0.29661326	0.868611705
**Evening Cortisol (µg/dl)**	0.727886104		0.625056613	0.337872161
**Melatonin (pg/ml)**	0.29661326	0.625056613		0.088240905
**Alpha-amylase (U/ml)**	0.868611705	0.337872161	0.088240905	

Men

Spearman r	Morning Cortisol (µg/dl)	Evening Cortisol (µg/dl)	Melatonin (pg/ml)	Alpha-amylase (U/ml)
**Morning Cortisol (µg/dl)**	1	-0.115854668	**-0.400260272**	0.086154558
**Evening Cortisol (µg/dl)**	-0.115854668	1	0.083929524	0.159095046
**Melatonin (pg/ml)**	-0.400260272	0.083929524	1	0.022380939
**Alpha-amylase (U/ml)**	0.086154558	0.159095046	0.022380939	1

95% CI	Morning Cortisol (µg/dl)	Evening Cortisol (µg/dl)	Melatonin (pg/ml)	Alpha-amylase (U/ml)
**Morning Cortisol (µg/dl)**	1	-0.4501 to 0.2468	**-0.6219 to -0.1192**	-0.2145 to 0.3719
**Evening Cortisol (µg/dl)**	-0.4501 to 0.2468	1	-0.2769 to 0.4240	-0.2050 to 0.4845
**Melatonin (pg/ml)**	-0.6219 to -0.1192	-0.2769 to 0.4240	1	-0.2746 to 0.3155
**Alpha-amylase (U/ml)**	-0.2145 to 0.3719	-0.2050 to 0.4845	-0.2746 to 0.3155	1

P values	Morning Cortisol (µg/dl)	Evening Cortisol (µg/dl)	Melatonin (pg/ml)	Alpha-amylase (U/ml)
**Morning Cortisol (µg/dl)**		0.520847155	**0.005307634**	0.56474121

**Evening Cortisol (μg/dl)**	0.520847155		0.642383567	0.376510912
**Melatonin (pg/ml)**	0.005307634	0.642383567		0.881298819
**Alpha-amylase (U/ml)**	0.56474121	0.376510912	0.881298819	

Supplementary Figure S2. Correlation dot plot between salivary melatonin and cortisol. Grey dotted bars represent cutoff values (5.2 pg/ml for melatonin; 0.743 μg/dl for cortisol).

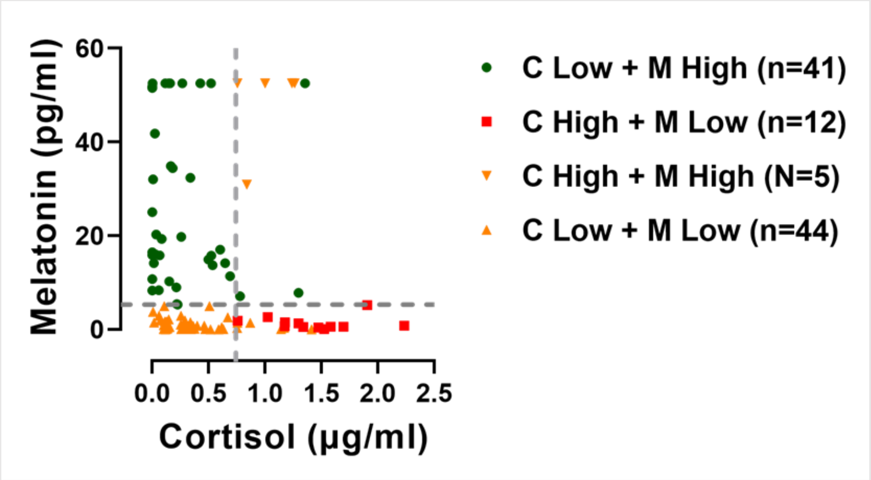

Supplementary Table S3. Correlation analyses among all psychodiagnostics questionnaires.

**All samples n=102**							
		**Stress Index**				**Sleep Index**	
95%CI		BDI	HAM-A	ERI	WAI	MEQ	PSQI
**Stress Index**	BDI	1					
	HAM-A	**0.70 to 0.85**	1				
	ERI	**0.18 to 0.53**	**0.21 to 0.55**	1			
	WAI	**-0.55 to -0.21**	**-0.52 to -0.17**	-0.29 to 0.09	1		
**Sleep index**	MEQ	-0.35 to 0.03	-0.34 to 0.04	-0.34 to 0.04	-0.15 to 0.24	1	
	PSQI	**0.38 to 0.67**	**0.54 to 0.76**	**0.08 to 0.45**	**-0.51 to -0.16**	**-0.40 to -0.02**	1
	ESS	**0.12 to 0.48**	**0.25 to 0.58**	**0.02 to 0.40**	-0.30 to 0.08	-0.27 to 0.12	-0.01 to 0.37
p-value		BDI	HAM-A	ERI	WAI	MEQ	PSQI
**Stress Index**	BDI						
	HAM-A	***< 0.001***					
	ERI	***< 0.001***	***< 0.001***				
	WAI	***< 0.001***	***< 0.001***	*0.296*			
**Sleep index**	MEQ	*0.091*	*0.119*	*0.119*	*0.645*		
	PSQI	***< 0.001***	***< 0.001***	***0.004***	***< 0.001***	***0.027***	
	ESS	***0.001***	***< 0.001***	***0.023***	*0.248*	*0.449*	*0.057*
**Women n=55**							
		**Stress Index**				**Sleep Index**	
95%CI		BDI	HAM-A	ERI	WAI	MEQ	PSQI
**Stress Index**	BDI	1					
	HAM-A	**0.66 to 0.87**	1				
	ERI	**0.16 to 0.62**	**0.29 to 0.69**	1			
	WAI	**-0.65 to -0.22**	**-0.63 to -0.18**	-0.40 to 0.12	1		
**Sleep index**	MEQ	-0.46 to 0.06	-0.46 to 0.05	-0.42 to 0.10	-0.16 to 0.37	1	
	PSQI	**0.17 to 0.62**	**0.35 to 0.73**	**0.02 to 0.52**	**-0.66 to -0.23**	-0.39 to 0.14	1
	ESS	**0.16 to 0.62**	**0.25 to 0.67**	**0.13 to 0.60**	-0.42 to 0.10	-0.36 to 0.17	-0.23 to 0.30
p-value		BDI	HAM-A	ERI	WAI	MEQ	PSQI
**Stress Index**	BDI						
	HAM-A	***< 0.001***					
	ERI	***0.001***	***< 0.001***				
	WAI	***< 0.001***	***0.001***	*0.28*			
**Sleep index**	MEQ	*0.114*	*0.107*	*0.196*	*0.422*		
	PSQI	***1.00E-03***	***< 0.001***	***0.027***	***< 0.001***	*0.321*	
	ESS	***0.001***	***< 0.001***	***0.003***	*0.209*	*0.455*	*0.784*

**Men n=47**							
		**Stress Index**				**Sleep Index**	
95%CI		BDI	HAM-A	ERI	WAI	MEQ	PSQI
**Stress Index**	BDI	1					
	HAM-A	**0.61 to 0.86**	1				
	ERI	**-0.01 to 0.54**	-0.08 to 0.48	1			
	WAI	**-0.54 to -0.01**	**-0.54 to 0.01**	-0.31 to 0.27	1		
**Sleep index**	MEQ	**-0.54 to -0.01**	**-0.54 to -0.01**	-0.44 to 0.12	-0.34 to 0.24	1	
	PSQI	**0.47 to 0.81**	**0.71 to 0.91**	-0.06 to 0.49	-0.48 to 0.07	**-0.58 to -0.06**	1
	ESS	-0.09 to 0.46	**0.12 to 0.62**	-0.26 to 0.32	-0.31 to 0.28	-0.30 to 0.28	**0.11 to 0.61**
p-value		BDI	HAM-A	ERI	WAI	MEQ	PSQI
**Stress Index**	BDI						
	HAM-A	***< 0.001***					
	ERI	***0.046***	*0.143*				
	WAI	***0.039***	***0.046***	*0.871*			
**Sleep index**	MEQ	***0.043***	***0.039***	*0.231*	*0.701*		
	PSQI	***< 0.001***	***< 0.001***	*0.111*	*0.134*	***0.016***	
	ESS	*0.173*	***0.004***	*0.851*	*0.947*	*0.961*	***0.006***

Abbreviations: BDI, Beck Depression Inventory, HAM-A, Hamilton Anxiety Scale, ERI, Effort Reward Imbalance, WAI, Work Ability Index, MEQ, Morning Evening Questionnaire, PSQI, Pittsburgh Sleep Quality Index, ESS, Epworth Sleepiness Scale. Correlation matrix including all samples (top), women (middle), and men (bottom). Top of each panel: 95% Confidence Intervals (CI), bottom of each panel: p values. Values are in bold when significant.

Supplementary Table S4. Frequencies relative to contingency analyses between work-related stress scales and salivary biomarkers.

**Total sample**	**BDI**		**HAM-A**		**E/R Ratio**		**WAI**	
	Absence N (%)	Presence N (%)	Absence N (%)	Presence N (%)	Absence N (%)	Presence N (%)	Good/ Excellent N (%)	Poor/ Moderate N (%)
Morning cortisol								
Low	76 (74.5)	9 (8.8)	63 (61.8)	22 (21.6)	70 (68.6)	15 (14.7)	74 (42.5)	11 (10.9)
High	12 (11.8)	5 (4.9)	11 (10.8)	6 (5.9)	15 (14.7)	2 (2.0)	14 (13.7)	3 (2.9)
Evening cortisol								
Low	55 (72.4)	14 (18.4)	44 (57.9)	25 (32.9)	54 (71.1)	15 (19.7)	55 (72.4)	14 (18.4)
High	7 (9.2)	0 (0.0)	7 (9.2)	0 (0.0)	7 (9.2)	0 (0.0)	7 (9.2)	0 (0.0)
Alpha-amylase								
Low	83 (81.4)	13 (12.7)	70 (68.6)	26 (25.5)	79 (77.5)	17 (16.7)	85 (83.3)	11 (10.8)
High	5 (4.9)	1 (1.0)	4 (3.9)	2 (2.0)	6 (5.9)	0 (0.0)	3 (2.9)	3 (2.9)
Melatonin								
Low	37 (36.3)	6 (5.9)	30 (29.4)	13 (12.7)	36 (35.3)	7 (6.9)	39 (38.2)	4 (3.9)
High	51 (50.0)	8 (7.8)	44 (43.1)	15 (14.7)	49 (48.0)	17 (16.7)	49 (48.0)	10 (9.8)
**Women**	**BDI**		**HAM-A**		**E/R Ratio**		**WAI**	
	Absence N (%)	Presence N (%)	Absence N (%)	Presence N (%)	Absence N (%)	Presence N (%)	Good/ Excellent N (%)	Poor/ Moderate N (%)
Morning cortisol								
Low	38 (69.1)	8 (14.5)	29 (52.7)	17 (30.9)	37 (67.3)	9 (16.4)	40 (72.7)	6 (10.9)
High	5 (9.1)	4 (7.3)	4 (7.3)	5 (9.1)	8 (14.5)	1 (1.8)	7 (12.7)	2 (3.6)
Evening cortisol								
Low	29 (67.4)	12 (27.9)	20 (46.5)	21 (18.9)	31 (72.1)	10 (23.3)	33 (76.7)	8 (18.6)
High	2 (4.7)	0 (0.0)	2 (4.7)	0 (0.0)	2 (4.7)	0 (0.0)	2 (4.7)	0 (0.0)
Alpha-amylase								
Low	41 (74.5)	11 (20.0)	32 (58.2)	20 (36.4)	42 (76.4)	10 (18.2)	46 (83.6)	6 (10.9)
High	2 (3.6)	1 (1.8)	1 (1.8)	2 (3.6)	3 (5.5)	0 (0.0)	1 (1.8)	2 (3.6)
Melatonin								
Low	22 (40.0)	5 (9.1)	17 (30.9)	10 (18.2)	23 (41.8)	4 (7.3)	25 (15.5)	2 (3.6)
High	21 (38.2)	7 (12.7)	16 (29.1)	12 (21.8)	22 (40.0)	6 (10.9)	22 (40.0)	6 (10.9)
**Men**	**BDI**		**HAM-A**		**E/R Ratio**		**WAI**	
	Absence N (%)	Presence N (%)	Absence N (%)	Presence N (%)	Absence N (%)	Presence N (%)	Good/ Excellent N (%)	Poor/ Moderate N (%)
Morning cortisol								
Low	38 (80.9)	1 (2.1)	34 (72.3)	5 (10.6)	33 (70.2)	6 (12.8)	34 (72.3)	5 (10.6)
High	7 (14.9)	1 (2.1)	7 (14.9)	1 (2.1)	7 (14.9)	1 (2.1)	7 (14.9)	1 (2.1)
Evening cortisol								
Low	26 (78.9)	2 (6.1)	24 (72.7)	4 (12.1)	23 (69.7)	5 (15.2)	22 (66.7)	6 (18.2)
High	5 (15.2)	0 (0.0)	5 (15.2)	0 (0.0)	5 (15.2)	0 (0.0)	5 (15.2)	0 (0.0)
Alpha-amylase								
Low	42 (89.4)	2 (4.3)	38 (80.9)	6 (12.8)	37 (78.7)	7 (14.9)	39 (83.0)	5 (10.6)
High	3 (6.4)	0 (0.0)	3 (6.4)	0 (0.0)	3 (6.4)	0 (0.0)	2 (4.3)	1 (2.1)
Melatonin								
Low	15 (31.9)	1 (2.1)	13 (27.7)	3 (6.4)	13 (27.7)	3 (6.4)	14 (29.8)	2 (4.3)
High	30 (63.8)	1 (2.1)	28 (59.6)	3 (6.4)	27 (57.4)	4 (8.5)	27 (57.4)	4 (8.5)

Supplementary Table S5. Frequencies relative to contingency analyses between sleep perceived quality scales and salivary biomarkers.

**Total sample**	**PSQI**		**ESS**	
	Good sleep quality N (%)	Poor sleep quality N (%)	Absence of daytime sleepiness N (%)	Presence of daytime sleepiness N (%)
Morning cortisol				
Low	65 (63.7)	20 (19.6)	75 (73.5)	10 (9.8)
High	12 (11.8)	5 (4.9)	12 (11.8)	5 (4.9)
Evening cortisol				
Low	52 (68.4)	17 (22.4)	58 (76.3)	11 (14.5)
High	6 (7.9)	1 (1.3)	6 (7.9)	1 (1.3)
Alpha-amylase				
Low	72 (70.6)	24 (23.5)	81 (79.4)	15 (14.7)
High	5 (4.9)	1 (1.0)	6 (5.9)	0 (0.0)
Melatonin				
Low	31 (30.4)	12 (11.8)	38 (37.3)	5 (4.9)
High	46 (45.1)	13 (12.7)	49 (48.0)	10 (9.8)
**Women**	**PSQI**		**ESS**	
	Good sleep quality N (%)	Poor sleep quality N (%)	Absence of daytime sleepiness N (%)	Presence of daytime sleepiness N (%)
Morning cortisol				
Low	35 (63.6)	11 (20.0)	40 (72.7)	6 (10.9)
High	6 (10.9)	3 (5.5)	6 (10.9)	3 (5.5)
Evening cortisol				
Low	30 (69.8)	11 (25.6)	33 (76.7)	8 (18.6)
High	2 (4.7)	0 (0.0)	2 (4.7)	0 (0.0)
Alpha-amylase				
Low	39 (70.9)	13 (23.6)	43 (78.2)	9 (16.4)
High	2 (3.6)	1 (1.8)	3 (5.5)	0 (0.0)
Melatonin				
Low	20 (36.4)	7 (12.7)	24 (43.6)	3 (5.5)
High	21 (38.2)	7 (12.7)	22 (40.0)	6 (10.9)
**Men**	**PSQI**		**ESS**	
	Good sleep quality N (%)	Poor sleep quality N (%)	Absence of daytime sleepiness N (%)	Presence of daytime sleepiness N (%)
Morning cortisol				
Low	30 (63.8)	9 (19.1)	35 (74.5)	4 (8.5)
High	6 (12.8)	2 (4.3)	6 (12.8)	2 (4.3)
Evening cortisol				
Low	22 (66.7)	6 (18.2)	25 (75.8)	3 (9.1)
High	4 (12.1)	1 (3.0)	4 (12.1)	1 (3.0)
Alpha-amylase				
Low	33 (70.2)	11 (23.4)	38 (80.9)	6 (12.8)
High	3 (6.4)	0 (0.0)	3 (6.4)	0 (0.0)
Melatonin				
Low	11 (23.4)	5 (10.6)	14 (29.8)	2 (4.3)
High	25 (53.2)	6 (12.9)	27 (57.4)	4 (8.5)
